# Rpd3L Contributes to the DNA Damage Sensitivity of *Saccharomyces cerevisiae* Checkpoint Mutants

**DOI:** 10.1534/genetics.118.301817

**Published:** 2018-12-17

**Authors:** Belén Gómez-González, Harshil Patel, Anne Early, John F. X. Diffley

**Affiliations:** * Chromosome Replication Laboratory, The Francis Crick Institute, NW1 1AT London, UK; † Bioinformatics and Biostatistics, The Francis Crick Institute, NW1 1AT London, UK

**Keywords:** DNA damage checkpoint, replication fork arrest, Rpd3L, histone deacetylase, replication

## Abstract

DNA replication forks that are stalled by DNA damage activate an S-phase checkpoint that prevents irreversible fork arrest and cell death. The increased cell death caused by DNA damage in budding yeast cells lacking the Rad53 checkpoint protein kinase is partially suppressed by deletion of the *EXO1* gene. Using a whole-genome sequencing approach, we identified two additional genes, *RXT2* and *RPH1*, whose mutation can also partially suppress this DNA damage sensitivity. We provide evidence that *RXT2* and *RPH1* act in a common pathway, which is distinct from the *EXO1* pathway. Analysis of additional mutants indicates that suppression works through the loss of the Rpd3L histone deacetylase complex. Our results suggest that the loss or absence of histone acetylation, perhaps at stalled forks, may contribute to cell death in the absence of a functional checkpoint.

THE process of DNA replication must ensure accurate chromosome duplication, even when contending with damaged DNA templates. Replication and DNA repair, take place on chromatin templates and, as a consequence, are influenced by chromatin remodelers and covalent histone modifications. The ability of cells to sense and respond appropriately to conditions that affect replication fork progression is crucial to maintain cell viability and genome integrity (Ciccia and Elledge 2010; Labib and De Piccoli 2011; Zeman and Cimprich 2014). The “checkpoint” that senses DNA damage and stalled DNA replication forks functions as an important anticancer barrier (Bartkova *et al.* 2005; Gorgoulis *et al.* 2005). Many anticancer drugs work by interfering with DNA replication and their efficacy may, therefore, be influenced by the checkpoint status of the cancer. Thus, understanding the processes that maintain stable replication forks through chromatin may contribute to improving therapies.

In *Saccharomyces cerevisiae*, as in multicellular eukaryotes, DNA damage checkpoints involve DNA damage detection, signal amplification, and signal transduction directed through phosphoinositide 3-kinase-related protein kinases (PIKK; Mec1 and Tel1 in yeast) that activate effector kinases (Rad53, Chk1, and Dun1 in yeast). Checkpoint kinases regulate many aspects of cell metabolism in response to DNA damage (Zegerman and Diffley 2009). These include blocking cell cycle progression (Allen *et al.* 1994), suppressing late origin firing (Santocanale and Diffley 1998; Shirahige *et al.* 1998; Santocanale *et al.* 1999; Lopez-Mosqueda *et al.* 2010; Zegerman and Diffley 2010), altering global gene expression patterns (Zhou and Elledge 1993; Allen *et al.* 1994; Huang *et al.* 1998), preventing irreversible DNA replication fork arrest (Lopes *et al.* 2001; Tercero and Diffley 2001; Tercero *et al.* 2003), and upregulating deoxyribonucleoside triphosphate (dNTP) levels (Desany *et al.* 1998; Zhao *et al.* 1998). The regulation of dNTP levels by the DNA damage checkpoint is essential for cell survival, even in the absence of DNA damage. Thus, the survival of *mec1*∆ or *rad53*∆ checkpoint mutants requires conditions in which dNTP levels have been elevated, such as deletion of the ribonucleotide reductase (RNR) inhibitor Sml1 (Zhao *et al.* 1998). However, this is not sufficient to support viability in the presence of DNA damage, such as that caused by methyl methanesulfonate (MMS) or hydroxyurea (HU). We have previously shown that replication forks in *rad53*∆ and *mec1*∆ mutant strains are unable to progress through damaged DNA, and that reinstating the checkpoint after fork stalling cannot rescue fork progression defects. We proposed that this irreversible replication fork arrest is one of the primary reasons for genotoxicity in these strains (Tercero and Diffley 2001; Tercero *et al.* 2003).

Despite its potential importance, we do not understand the nature of the irreversible fork catastrophe in checkpoint mutants, nor do we know how the DNA damage checkpoint protects forks from this fate. Chromatin immunoprecipitation experiments suggested that the checkpoint might prevent loss of replisome components from the fork (Lucca *et al.* 2004; Cobb *et al.* 2005), but more recent work has shown that stalled replisomes appear to be stable even in the absence of a functional checkpoint, both in yeast and human cells (De Piccoli *et al.* 2012; Dungrawala *et al.* 2015). We have taken an unbiased genetic approach to further understand why checkpoint mutant cells are hypersensitive to DNA damage, revealing a connection between chromatin state and replication fork stability.

## Materials and Methods

### Strains and plasmids

All strains used are listed in Supplemental Material, Table S1 and are derived from W303 (*ade2-1 ura3-1 his3-11*, *15 trp1-1 leu2-3*, *112 can1-100*). Genes were deleted by gene replacements of the endogenous wild-type allele via genetic recombination with the corresponding drug-resistant cassettes, obtained by the PCR strategies described previously for *KANMX6* (Longtine *et al.* 1998), *NATMX6* (Van Driessche *et al.* 2005), *NATNT2*, and *HPHNT1* (Janke *et al.* 2004). Histone mutants were made by plasmid shuffle and checked by PCR, western blotting, and sequencing with plasmids carrying the wild-type and mutant H3K36R or H3K9R histone genes (pDM9, MBB286, and pWZ414-F53) that were previously described (Quan and Hartzog 2010).

*YEplac112*, *YEplac112-Rpd3*, and *YEp-lac112-rpd-3H150A* were previously described (Kadosh and Struhl 1998). The *pRS303-RPH1* plasmid was constructed as follows. The *RPH1* genomic fragment was amplified by PCR using Phusion DNA polymerase with oligonucleotides 5′-ATGCCATCCCGGGACACAAAAAAAGCCCTTATAAC-3′ and 5′-GCTGCACACTAGTTCAGTTTAAAGGTGTACTCTG-3′, digested with *Xma*I-*Spe*I, and cloned into *Xma*I-*Spe*I-cut *pRS303*. *rph1-H235A*, *rph1-S652A*, and *rph1-S652D* mutants were created by site-directed mutagenesis of *RPH1* and the *RPH1* gene was sequenced to verify correct substitution of the selected fragments. The *rph1*-wild-type and point mutant strains were created by targeting *Nhe*I-digested constructs *pRS303-RPH1*, *pRS303-rph1-H235A*, *pRS303-rph1-S652A*, or *pRS303-rph1-S652D* into the *HIS3* locus of an *rph1*∆ *rad53*∆ strain.

### Cell growth, media, and drug sensitivity assays

Cells were grown at 30° in YPD medium (1% yeast extract, 2% bacto peptone, and 2% glucose) unless indicated. When backcrossing the isolated checkpoint suppressors with the parental strain, we dissected tetrads and replicated the spores into media containing MMS and phloxine B plates to help distinguish the spores that were surviving MMS. To confirm the suppressor mutations, a minimum of two independent deletions were tested for each gene to exclude mutations that might have arisen during the deletion procedure. We also backcrossed the strain deleted for each tested gene with the parental one to obtain a heterozygous diploid and analyzed the cosegregation of the deletion with the suppressor phenotype after sporulation. In all cases that MMS was used in plates, MMS was added to the melted agar at the indicated concentration just before pouring the plates and the MMS-containing plates were dried with the lid in place for exactly 24-hr before use. Plates were sealed with parafilm during incubation. Dilution-plating assays were a 1:10 dilution series of cultures on the indicated plates and incubated for 3 days at 30°, or irradiated with ultraviolet (UV) radiation at the indicated dosages and then incubated for 3 days at 30°. For the MMS sensitivity assays in a single S phase, the indicated strains were released from G1 arrest at 30° in YPD medium containing 0.015% MMS, and cell survival was scored during α-factor arrest and at the indicated time points. Relative survival was calculated as the percentage of survival of each strain relative to the survival data of the *sml1*∆ control strain obtained on the same day. Cell cycle blocks with α-factor were as described previously (Diffley *et al.* 1994). Cycloheximide was used at a concentration of 100 μg/ml.

### Protein extracts

Proteins were extracted by TCA as described (Foiani *et al.* 1994) and were then loaded on a 15% acrylamide-bisacrylamide gel, dried, and exposed in a Fujifilm FLA-5000 phosphorimager.

### Whole-genome sequencing

DNA for whole-genome sequencing was extracted by the CTAB protocol as previously described (Moriel-Carretero and Aguilera 2010) and sheared using the Covaris S2 to ∼300 bp. The sheared DNA samples were end repaired, poly-A tailed, and Illumina Single-End Adapters were ligated (paired-end protocol; Illumina, San Diego, CA). The standard protocol by Illumina was adjusted to fit our samples. We used Agencourt AMPure XP beads (AMPure bead protocol; Beckman Coulter) at 0.8× ratio to size select out adapter dimers after adapter ligation. The Illumina kit Phusion enzyme was replaced by Kapa HiFi HotStart ready mix (Kapa Biosystems, Cape Town, South Africa). Post PCR, we used AMPure XP beads at a 1:1 ratio to maintain size integrity, which also helped us to optimize the pH and other salt concentrations to allow us to use the Invitrogen SizeSelect E-gel system (SizeSelect gel protocol; Life Technologies). Unlike for the Illumina paired-end sample preparation protocol, we ran the PCR before the gel. This improved visualization of the product and removal of the correct band. To aid us in removing any gel residue that would cause issues in later quality control (QC) and cluster formation, we purified with a QIAquick gel extraction kit (Gel purification protocol; QIAGEN, Valencia, CA). After a final QC step using the DNA 1000 BioAnalyser 2100 chip (Agilent, Santa Clara, CA), the libraries were ready for Flowcell cluster formation on a cluster station followed by Illumina paired-end sequencing on either the Genome Analyzer IIx or HiSeq 2500 with a read length of 36 and 101 bp, respectively. Due to the increased output of the HiSeq 2500, we were able to multiplex 17 samples per lane.

### Single-nucleotide variant calling in MMS-resistant suppressor strains

Paired-end reads were aligned to the yeast sacCer2 genome assembly using Novoalign 2.07.14 (http://novocraft.com), with the parameters for the mean and SD of the expected size distribution set to 175 and 125, respectively. Alignments for 101-bp paired-end reads were postprocessed for removal of reads that could have arisen from PCR duplication (picard-tools 1.81; http://sourceforge.net/projects/picard/). Approximately 96% of paired reads were found to align concordantly to the reference genome resulting in a median coverage of 200× across all samples.

Base-level nucleotide counts were obtained using deepSNV 1.2.3 (Gerstung *et al.* 2012) with a minimum base quality threshold of 30. Single-nucleotide variant (SNV) calling was performed simultaneously across the relevant sample groups per experiment using scripts written in Python. A minimum allele count of 5 and minimum allele frequency of 0.25 were applied for the *rad53*∆ *chk1*∆ *exo1*∆ (S64Bα *vs.* W64Aα strain) and *exo1*∆ *rad53*∆ (YMS6 *vs.* YGDP939 strain) genome sequencing experiments. These thresholds were increased to a minimum allele count of 20 and minimum allele frequency of 0.40 for the *rad53*∆ batch sequencing experiment (MMS-resistant, suppressor-containing strains *vs.* the YJT75 strain). Multiallelic loci and variants identified in any of the control samples with an allele frequency of 0.1 or above were discarded. Variant annotation was performed with snpEff 3.0j (Cingolani *et al.* 2012) using the sacCer2.61 Ensembl genomic database provided by the package.

SNVs that passed the above criteria were further filtered to only include those that were labeled as nonsynonymous mutations and had a minimum allele frequency of 0.7. Among the top 10 genes that were found to have a recurring missense mutation, 14 mutations were found in genes involved in nonsense-mediated mRNA decay (*NMD2*, *DCP2*, *XRN1*, and *UPF3*). Thus, we tested the deletions of a total of 32 nonessential genes related to RNA and DNA metabolism (*NMD2*, *XRN1*, *UPF3*, *SIR1*, *SIR4*, *ESC1*, *PPH22*, *MOT2*, *CTF18*, *SAC3*, *RAI1*, *HRB1*, *SUM1*, *YKU70*, *TDA7*, *MLP2*, *SPT7*, *DAS1*, *VPS72*, *STB6*, *RTT106*, *BMH2*, *MPT5*, *DEF1*, *LCL3*, *HFM1*, *ARP8*, *NUP100*, *SWD1*, *RIF1*, *BDF2*, and *NUP170)* and identified three pathways: nonsense-mediated mRNA decay, silencing of the mating-type loci *HMR* and *HML* (HM loci), and the Rpd3L histone deacetylase (HDAC) complex.

If we apply a less-stringent minimum allele frequency (0.4 instead of 0.7) and consider mutations in Rph1, Rpd3L components (as defined for Rpd3L-expanded, Shevchenko *et al.* 2008), chromatin silencing [defined as the gene ontology (GO) processes of “chromatin silencing at silent mating-type cassette,” “chromatin-silencing complex,” or “negative regulation of gene silencing”], mRNA decay (defined as the GO term “nuclear-transcribed mRNA catabolic process, nonsense-mediated decay”), or MMS hyperresistance, we can assign a mutation to 63 out of the 95 suppressors sequenced. Given that we did not analyze mutations mapping outside ORFs, which might affect also the gene expression levels of genes related to these pathways, and that there are many possible unknown factors that can affect chromatin acetylation, chromatin silencing, or MMS resistance, probably most of the 95 suppressors sequenced can be explained by one of the three identified pathways, although we cannot rule out other pathways.

### Data availability

Strains and plasmids are available upon request. The authors affirm that all data necessary for confirming the conclusions of the article are present within the article, figures, and tables. All raw sequence data and processed SNV calls can be accessed with Gene Expression Omnibus accession GSE113869. Supplemental material available at Figshare: https://doi.org/10.25386/genetics.7473365.

## Results

### Loss of *EXO1* and *RXT2* suppresses the DNA damage sensitivity of *rad53*∆ cells

**Figure 1:**
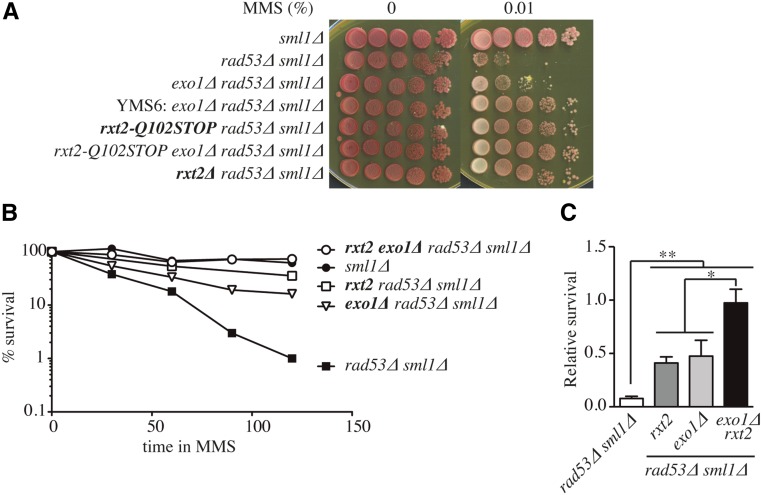
Identification of *rxt2* as an extragenic suppressor of the MMS sensitivity of checkpoint mutants. (A) Suppression of the *rad53*∆ sensitivity to chronic MMS treatment by *exo1*∆, *rxt2-Q102STOP*, *rxt2*∆, and double mutants. Strains: YJT72, YJT75, YGDP939, YMS6, 938rxt2, 939rxt2, and YBG628 (B) Suppression of the *rad53*∆ sensitivity to MMS by *rxt2-Q102STOP*, *exo1*∆, and double mutants in a single S phase was studied by treating the indicated strains with 0.015% MMS for the indicated time points after release from G1 synchronization, and scoring cell survival by colony-forming units after plating in fresh media. The mean of at least three experiments is shown. Strains: YBG022, YBG023, YBG245, YBG026, and 939rxt2. (C) Relative survival of S-phase cells treated with MMS 0.015% for 2 hr with respect to the survival of the *sml1*∆ control strain (YBG022). Strains: YBG023, YBG245, YBG026, and 939rxt2. The mean and SEM of at least three experiments is shown. * *P* < 0.05 and ** *P* < 0.005 (Student’s unpaired *t*-test).

We previously reported that deletion of the *EXO1* gene can suppress the sensitivity of *rad53*∆ cells to MMS (Segurado and Diffley 2008). However, we became aware that the striking resistance to MMS seen in one of our published *rad53*∆ *exo1*∆ strains (YMS6) was not seen in other genetic backgrounds (K. Labib and G. De Piccoli, personal communication). This led us to consider that additional mutations in YMS6 may have contributed to its heightened MMS resistance. To test this, we sequenced the genome of YMS6 and compared it to a newly generated *exo1*∆ *rad53*∆ strain (YGDP939), which did not show such pronounced MMS resistance. We established criteria to define mutations (see *Materials and Methods* and Table S2) and we focused on mutations within ORFs that changed coding sequences. We found seven such mutations. Six were missense mutants, but one was a nonsense mutation in the *RXT2* gene (*rxt2-Q102STOP*), which truncated the last 329 amino acid residues of the encoded protein (Table S2). We remade this truncation; Figure 1A shows that, like the original mutant, this reconstructed *rxt2-Q102STOP* partially suppresses the MMS sensitivity of *rad53*∆. Furthermore, complete deletion of the *RXT2* gene conferred similar MMS resistance (Figure 1A). To examine the effect of *RXT2* and *EXO1* deletion on viability in a single cell cycle, cells from various strains were synchronized in G1 phase with α-factor mating pheromone and released from the G1 arrest in the presence of MMS. As shown in Figure 1B, *rxt2-Q102STOP* partially restored the viability lost by *rad53*∆ upon passage through S phase in the presence of MMS, indicating that wild-type Rxt2 contributes to cell death in *rad53*∆ cells treated with MMS even in a single S phase. Therefore, both *rxt2-Q102STOP* and *exo1*∆ can suppress the DNA damage sensitivity of *rad53*∆ cells individually, with *rxt2-Q102STOP* being a better growth suppressor under chronic MMS treatment. The suppression by *rxt2-Q102STOP* and *exo1*∆ were additive, indicating that they are in different pathways (Figure 1C). Thus, mutation/loss of both *EXO1* and *RXT2* contributed to the suppression of lethality in MMS previously seen in YMS6 (Segurado and Diffley 2008). Our previous conclusion that deletion of *EXO1* suppresses irreversible fork arrest in *rad53*∆ cells is likely unaffected by this discovery, since the strains used in the replication fork stability experiments were derived separately from YMS6 and showed MMS sensitivity similar to YGDP939. Moreover, neither viability nor irreversible fork arrest in the *rad53*∆ *chk1*∆ strain and the *mec1*∆ strain were suppressed by deletion of *EXO1*, consistent with our previous conclusion that these other checkpoint kinases have separate roles in fork stabilization. Nonetheless, the results presented above identified a new Rad53 suppressor (*rxt2*) and suggested that multiple pathways might contribute to the DNA damage sensitivity of *rad53* mutants.

### The Rpd3L HDAC contributes to DNA damage sensitivity in *rad53*∆ cells

**Figure 2:**
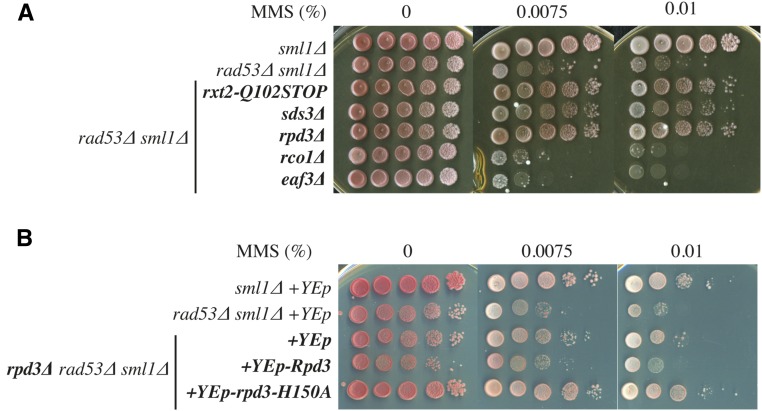
The loss of Rpd3L histone deacetylase suppresses the MMS sensitivity of checkpoint mutants. (A) The nonsense mutation *rxt2-Q102STOP*, and deletions in Rpd3 or the Rpd3L-specific component Sds3, but not the Rpd3S-specific component Rco1 or Eaf3, suppress the *rad53*∆ sensitivity to MMS. Strains: YJT72, YJT75, YBG245, YBG615, YBG369, YBG618, and YBG616. (B) Expression of Rpd3, but not rpd3-H150A histone deacetylase catalytically dead mutant, prevents the suppression by *rpd3*∆. Strains: YJT72+YEplac112, YJT75+YEplac112, YBG369+YEplac112, YBG369+YEplac112-Rpd3, and YBG369+YEplac112-rpd3-H150A.

Rxt2 is a subunit of Rpd3L, a HDAC complex whose catalytic subunit is Rpd3. Rpd3L is in “balance” with another HDAC complex, Rpd3S (Carrozza *et al.* 2005), which shares several subunits with Rpd3L, including Rpd3. However, each complex also contains several specific subunits (Carrozza *et al.* 2005). Whereas Rpd3L localizes primarily to promoter regions to repress transcription, Rpd3S is recruited at the 3′-end of transcribed regions (Joshi and Struhl 2005). To address whether the suppression was due to a defect in Rpd3L HDAC, we studied the effect of deleting other Rpd3L components on the MMS sensitivity of *rad53*∆. Both *sds3*∆ and *rpd3*∆ suppressed the MMS sensitivity of *rad53*∆ as well as *rxt2-Q102STOP* (Figure 2A). However, the deletion of Rpd3S-specific components, such as Eaf3 or Rco1, did not suppress (Figure 2A). Therefore, suppression is specific to the loss of Rpd3L.

We sought to determine if Rpd3 catalytic activity was involved in the suppression. For this purpose, we expressed Rpd3 and an Rpd3-H150A HDAC catalytic mutant (Kadosh and Struhl 1998) in our *rpd3*∆ *rad53*∆ *sml1*∆ background. As shown in Figure 2B, expression of Rpd3, but not Rpd3-H150A, prevented the suppression, making cells sensitive to MMS.

The deletion of *RPD3* had previously been shown to suppress the sensitivity to UV and HU of *rad9* and *mec1* checkpoint mutants. This suppression was shown to be functional spindle assembly checkpoint-dependent (Scott and Plon 2003). However, we found that deletion of the spindle assembly checkpoint gene *MAD1* had no effect on the suppression of MMS sensitivity in *rad53*∆ cells by *rpd3*∆ (Figure S1), indicating that the suppression was independent of the spindle assembly checkpoint. Furthermore, although Mec1- and Tel1-dependent cross talk between the DNA damage and the spindle assembly checkpoint has been suggested (Kim and Burke 2008), deletion of *TEL1* had no effect on the suppression by *rpd3*∆ (Figure S1).

### Identification of *RPH1* deletion as a new checkpoint suppressor

**Figure 3:**
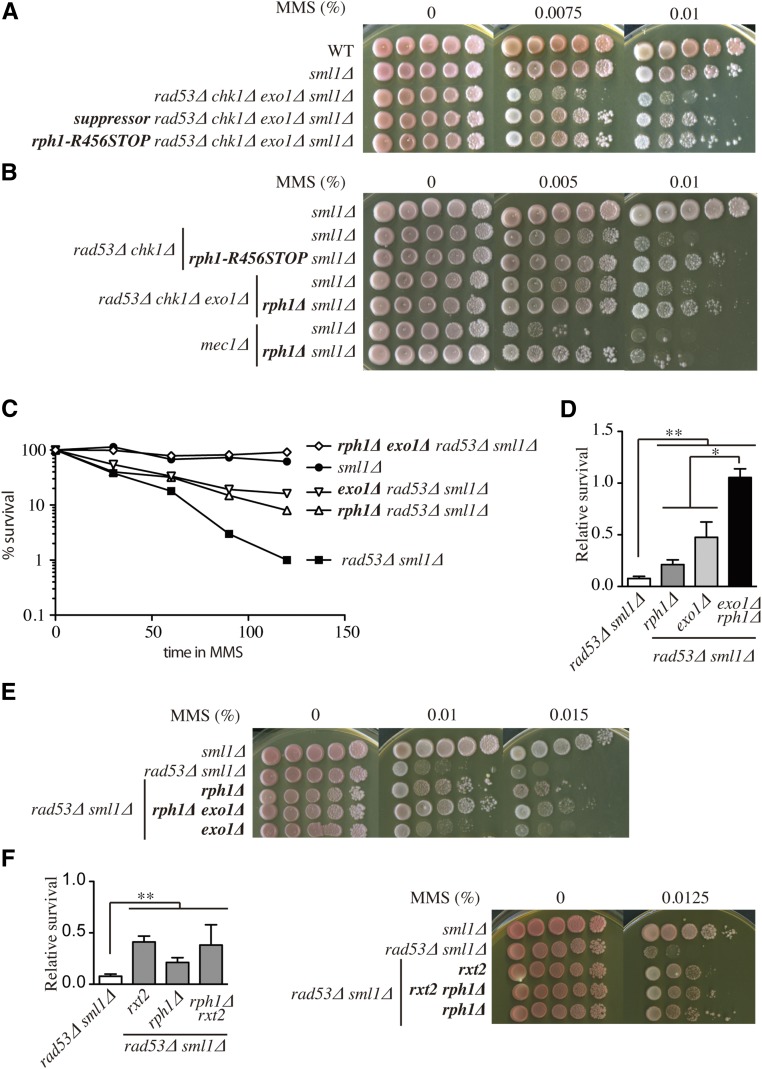
Identification of *rph1* as an extragenic suppressor of the MMS sensitivity of checkpoint mutants. (A) Suppression of the *rad53*∆ *chk1*∆ *exo1*∆ sensitivity to MMS by the nonsense mutation *rph1-R456STOP*. Strains: W303-1A, YJT72, W64Aα, S64Bα, and YBG617. (B) *rph1*∆ suppression of the sensitivity to MMS of *rad53*∆ *chk1*∆, *rad53*∆ *chk1*∆ *exo1*∆, and *mec1*∆. Strains: YJT72, YBG611, 2609CRS, YMS167, W6RPH1∆1, YJT74, and YBG612. (C) Suppression of the *rad53*∆ sensitivity to MMS by *rxt2-Q102STOP*, *exo1*∆, and double mutants in a single S phase, as in Figure 1B. Strains: YBG023, YBG034, YBG026, and YBG032. (D) Relative survival of S-phase cells as in Figure 1C. * *P* < 0.05 and ** *P* < 0.005 (Student’s unpaired *t*-test). Strains: YBG022, YBG023, YBG034, YBG026, and YBG032. (E) Suppression of the *rad53*∆ sensitivity to chronic MMS treatment by *exo1*∆, *rph1*∆, and double mutants. Strains: YBG022, YBG023, YBG034, YBG026, and YBG032. (F) Epistatic suppression of the *rad53*∆ sensitivity to a single cell cycle (left panel) and chronic MMS treatment (right panel) by *rxt2-Q102STOP* and *rph1*∆. * *P* < 0.05 and ** *P* < 0.005 (Student’s unpaired *t*-test). Strains: YBG022, YBG023, YBG245, YBG034, and YBG348. WT, wild-type.

To further understand how checkpoints promote viability in the presence of DNA damage, we were interested in identifying other suppressors in an unbiased manner. Given that our previous report (Segurado and Diffley 2008) identified *exo1*∆ as a suppressor of the DNA damage sensitivity of *rad53*∆ but not *rad53*∆ *chk1*∆ mutant cells, we isolated and characterized colonies from *rad53*∆ *chk1*∆ *exo1*∆ cells that survived on plates containing MMS. One such suppressor was backcrossed five times with the parental strain prior to whole-genome sequencing (see *Materials and Methods* and Table S3). Among the 12 nonsilent mutations in ORFs, we only found one mutation that caused a protein truncation: a nonsense mutation in the *RPH1* gene causing a deletion of the last 340 amino acids of the 796-amino acid Rph1 protein (*rph1-R456STOP*). We remade this truncation in an unmutagenised genetic background and showed that the *rph1-R456STOP* truncation promoted levels of survival in the *rad53*∆ *chk1*∆ *exo1*∆ background very similar to those of the strain carrying the original suppressor (Figure 3A). *rph1-R456STOP* also suppressed the sensitivity of *rad53*∆ cells to other DNA-damaging agents including UV radiation, camptothecin (CPT), and HU, but this suppression was never as striking as that seen in MMS (Figure S2A). The complete deletion of the *RPH1* gene also promoted the resistance of *rad53*∆ *chk1*∆ *exo1*∆ cells to DNA damage (Figure 3B). Moreover, deletion of *RPH1* partially suppressed the MMS sensitivity of *rad53*∆ (Figure 3E), *rad53*∆ *chk1*∆, and *mec1*∆ checkpoint mutant strains (Figure 3B). Deletion of *RPH1* also partially suppressed MMS sensitivity within a single S phase in an α-factor block and release experiment (Figure 3, C and D).

The deletion of *EXO1* improved the survival of *rph1*∆ *rad53*∆ both in a single-cell cycle experiment (Figure 3, C and D) and upon chronic exposure to MMS (Figure 3E) indicating that, as in the case of Exo1 and Rxt2, Exo1 and Rph1 also act in separate pathways. We wondered whether the suppression caused by *rph1* was related to that caused by Rpd3L loss. As shown in Figure 3F, Rxt2 and Rph1 were epistatic for the suppression, indicating that they act in the same pathway.

### Loss of Rph1 demethylase activity is not sufficient for suppression

**Figure 4:**
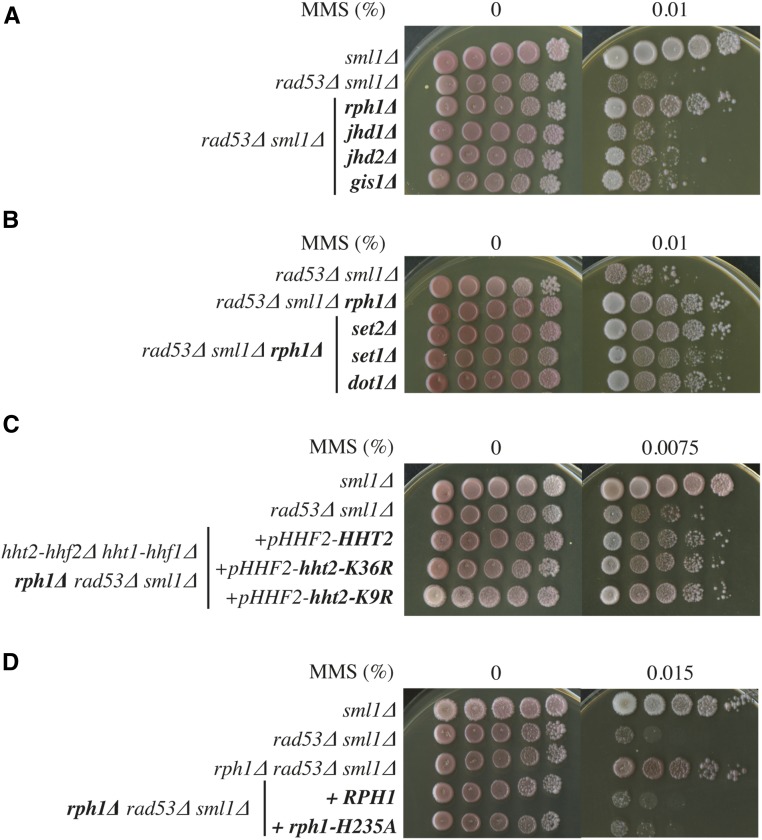
The suppression by *rph1*∆ is not caused by the lack of Rph1 demethylase activity. (A) Deletion of Jhd1, Jhd2, or Gis1 does not suppress the MMS sensitivity of *rad53*∆. Strains: YBG022, YBG023, YBG034, YBG038, YBG130, and YBG469. (B) Deletion of Set2, Set1, or Dot1 does not prevent the suppression of the MMS of *rad53*∆ by *rph1*∆. Strains: YBG023, YBG034, YBG607, YBG608, and YBG609. (C) H3-K36R or H3K9R mutants cannot prevent the suppression of the MMS of *rad53*∆ by *rph1*∆. Strains: YBG047, YBG610, YBG101, YBG154, and YBG153. (D) The expression of a wild-type or demethylase-deficient Rph1 prevents the suppression by *rph1*∆. Either wild-type or mutated Rph1 versions were integrated in the genome of a *rad53*∆ *rph1*∆ strain. Strains: YBG022, YBG023, YBG034, YBG475, and YBG500.

Rph1 is a JmjC domain-containing histone demethylase. Deletion of the other known histone demethylases (Jhd1, Jhd2, or Gis1) did not show any suppression of the *rad53*∆ sensitivity to MMS (Figure 4A), arguing that the suppression is specific for the loss of Rph1. Rph1 demethylates histone H3 lysine 36 (H3K36) and is the only known demethylase for the trimethylated state of H3K36. If suppression was due to an accumulation of H3K36 methylation, it should require the only known H3K36 methyltransferase, Set2. However, as shown in Figure 4B, *rph1*∆-induced suppression of the *rad53*∆ sensitivity to MMS still occurred in *set2*∆ cells. Rph1 can also demethylate H3K9 *in vitro*, although this modification has not been detected in yeast *in vivo* (Klose *et al.* 2007). To exclude the role of H3K36 or H3K9 methylation, we constructed strains in which all copies of the histone H3 and H4 genes (*HHT1*, *HHT2*, *HHF1*, and *HHF2*) were deleted from the genome and supplemented by a plasmid with a single copy of *HHF*, and either a wild-type copy of *HHT* or the nonmethylatable *hht2-K36R* or *hht2-K3K9R* mutants (Quan and Hartzog 2010). As shown in Figure 4C, neither *hht2-K36R* nor *hht2-K3K9R* prevented the suppression caused by *rph1*∆. To address if any other histone methylation was responsible for the suppression, we eliminated the other two known histone methyltransferases, Set1 and Dot1, with no effect on *rph1*∆-induced suppression either (Figure 4B). These experiments show that *rph1*∆-induced suppression of the *rad53*∆ sensitivity to MMS is not caused by the histone demethylase function of Rph1. To test the possibility that suppression might be due to demethylation of some nonhistone substrate, we constructed strains expressing either the wild-type *RPH1* gene or a catalytic-deficient *rph1-H235A* mutant, which disrupts Rph1 demethylase activity (Liang *et al.* 2011). In each case, expression was driven by the *RPH1* promoter at the *HIS3* locus in a *rph1*∆ *rad53*∆ background. As shown in Figure 4D, both the wild-type *Rph1* and *rph1-H235A* demethylase mutant fully prevented the suppression caused by *rph1*∆. Therefore, the elimination of the Rph1 demethylase function is not sufficient to suppress the MMS sensitivity of *rad53*∆, indicating that Rph1 must have a function independent of its demethylase activity.

### Identification of additional suppressor pathways

Finally, we asked whether additional pathways might contribute to suppression. We isolated 100 independent *rad53*∆ *sml1*∆ suppressors from MMS plates and confirmed their resistance to MMS. We then performed whole-genome sequencing on the 95 remaining MMS-resistant suppressors (see *Materials and Methods* and Table S4). By focusing on nonsilent coding mutations, of which there was an average of 14 per strain, and, in particular, nonsense mutations of which there were a total of 43 across the 95 mutant strains, we were able to prioritize mutations for retesting. We found *RPH1*, *RPD3*, and *SAP30* mutated in 4 of our 95 suppressors (Table S4), though we did not find *RXT2* or the remaining Rpd3L subunits, indicating that the screen was not saturated. We initially chose 32 genes mutated in the suppressor strains, which had been implicated in nucleic acid metabolism to test further (see *Materials and Methods*). We found that the sensitivity to MMS of *rad53*∆ cells was suppressed by the deletion of 6 of the 31 candidates: the nonsense-mediated mRNA decay (NMD) factors *NMD2*, *UPF3*, and *XRN1*, and the chromatin-silencing factors *SIR1*, *SIR4*, and *ESC1*.

**Figure 5:**
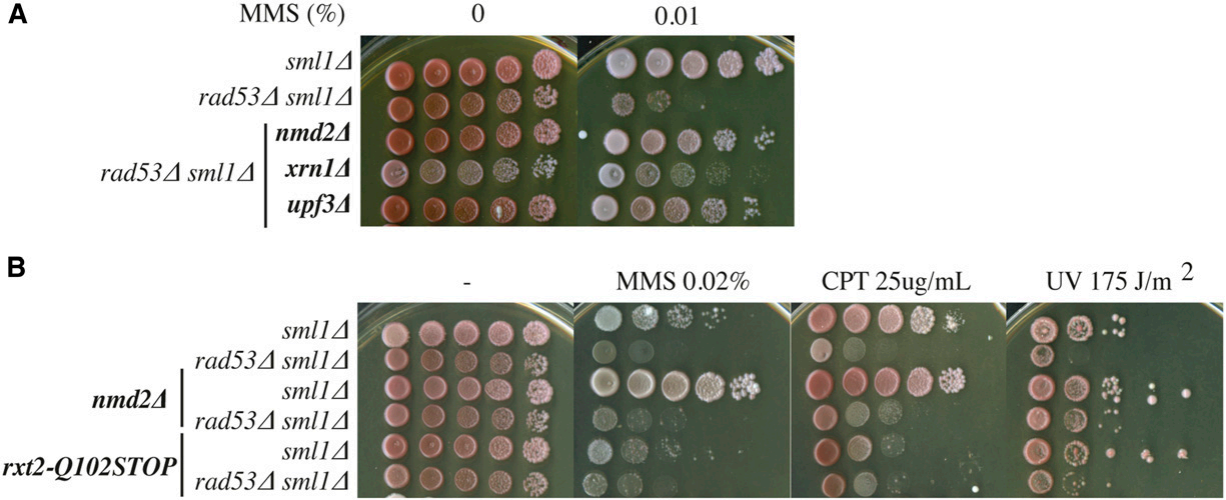
The deletion of NMD factors suppresses the *rad53*∆ sensitivity to MMS due to general hyperresistance to DNA damage. (A) Deletions of the NMD factors Nmd2, Xrn1, or Upf3 suppress the *rad53*∆ sensitivity to MMS. Strains: YJT72, YJT75, YBG071, YBG076, and YBG121. (B) *nmd2*∆, but not *rxt2-Q102STOP*, is hyperresistant to MMS, CPT, and UV. Strains: YJT72, YJT75, YBG455, YBG071, YBG456, and YBG245. CPT, camptothecin; NMD, nonsense-mediated mRNA decay; MMS, methyl methanesulfonate; UV, ultraviolet.

*nmd2*∆ and *upf3*∆ strongly suppress the MMS sensitivity of *rad53*∆ cells, while *xrn1*∆ can also suppress, despite the fact that *xrn1*∆ mutants have a growth defect (Kipling *et al.* 1991) (Figure 5A). *nmd2*∆ also caused slightly increased resistance to other DNA-damaging agents such as CPT or UV (Figure 5B). However, we found that deletion of *NMD2* also significantly enhanced the survival of *RAD53* cells in MMS, indicating that this effect is not checkpoint-specific (Figure 5B) and suggesting that deletion of NMD factors confers a general increased resistance to DNA damage.

**Figure 6:**
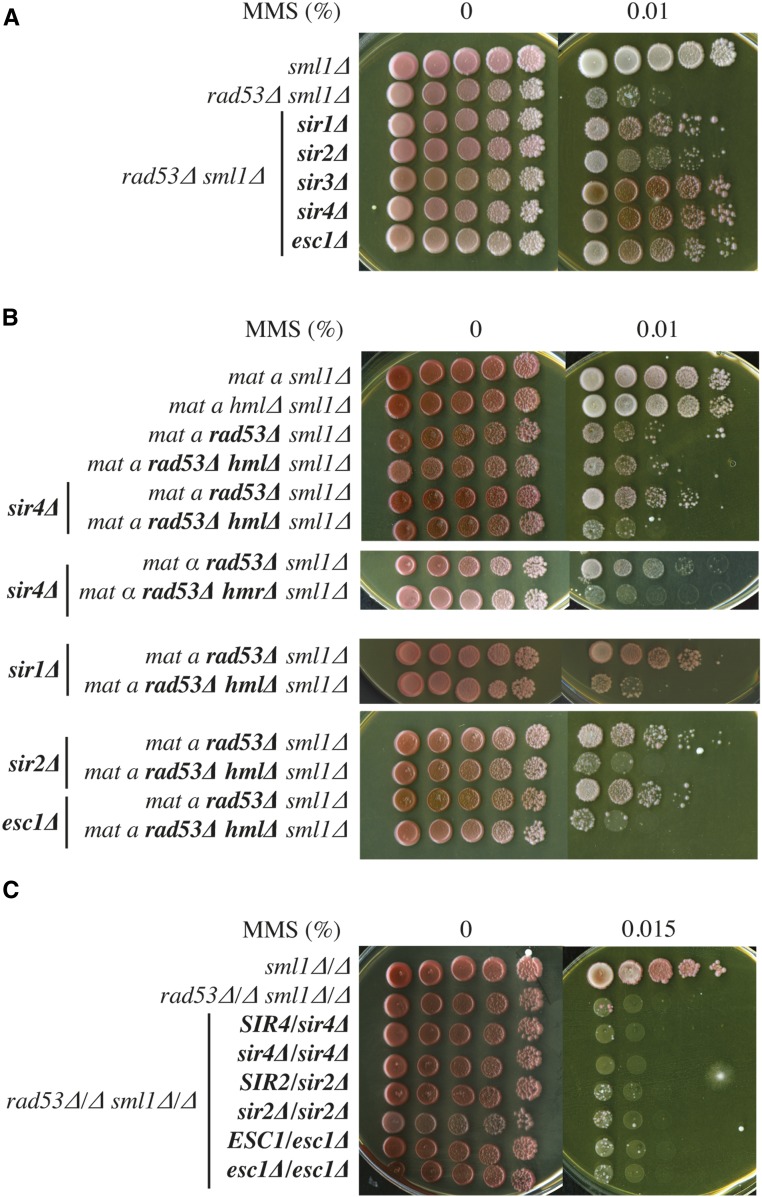
The deletion of silencing factors suppresses the *rad53*∆ sensitivity to MMS due to HM desilencing. (A) Deletion of the Sir1-4-silencing factors or Esc1 suppresses the *rad53*∆ sensitivity to MMS. Strains: YJT72, YJT75, YBG255, YBG241, YBG605, YBG604, and YBG606. (B) The suppression of the *rad53*∆ haploid sensitivity to MMS by *sir4*∆, *sir1*∆, *sir2*∆, or *esc1*∆ is dependent on the *HM* loci. Strains: YJT72, YBG400, YJT75, YBG239, YBG604, YBG265, YBG263, YBG268, YBG255, YBG626, YBG241, YBG355, YBG606, and YBG359. (C) The deletion of Sir2, Sir4, or Esc1 does not suppress the *rad53*∆ sensitivity to MMS in diploids. Strains: YBG200, YBG181, YBG264, YBG269, YBG354, YBG388, YBG358, and YBG385.

The finding that deletion of *SIR1*, *SIR4*, or *ESC1* suppressed the DNA damage sensitivity of *rad53*∆ cells was consistent with previous work (Hu *et al.* 2001), and suggested a role for chromatin silencing. We set out to understand how chromatin silencing may be involved in DNA damage sensitivity. In *S. cerevisiae*, chromatin silencing has been described at three separate groups of loci: the silent mating-type cassettes (*HML* and *HMR*), telomeres, and rDNA. Silencing at the HM loci requires all four *SIR* genes (*SIR1-4*), whereas telomere position effect requires *SIR2-4* and rDNA silencing requires only *SIR2* [reviewed in Rusche *et al.* (2003)]. Figure 6A shows that deletion of any one of the four silencing factors suppressed the MMS sensitivity of *rad53*∆. Since Sir1 has only been shown to be required for silencing at the HM loci, this suggested that the suppression might work through deregulated HM expression. Consistent with this, *MAT* heterozygosity has been shown to confer DNA damage hyperresistance (Friis and Roman 1968; Heude and Fabre 1993; Valencia-Burton *et al.* 2006).

If the suppression is due to inappropriate expression of HM loci, then the suppression due to *SIR* gene deletion should require the presence of the HM loci. As shown in Figure 6B, the deletion of *HML* in *MAT***a** cells fully prevented the suppression caused by *sir1*∆, *sir2*∆, *sir4*∆, and *esc1*∆. Similarly, deletion of *HMR* in *MAT*α cells fully prevented the suppression caused by *sir4*∆. HM derepression allows haploid yeast cells to express both **a**- and α-mating-type (*MAT*) genes, and thus, gene expression patterns are regulated as in diploids. Therefore, we predicted that the deletion of *SIR* or *ESC1* genes should not suppress the DNA damage sensitivity of *rad53*∆*/rad53*∆ diploids. As shown in Figure 6C, the enhanced MMS sensitivity of *rad53*∆*/rad53*∆ diploid cells was not suppressed when one or both *SIR2*, *SIR4*, or *ESC1* copies were deleted, confirming that the suppression caused by *sir2*∆, *sir4*∆, and *esc1*∆ is a consequence of the mating-type heterozygosity in haploids.

Because Rpd3 antagonizes Sir2-dependent silent chromatin propagation (Zhou *et al.* 2009) and the cellular pool of Sir factors is limiting (Maillet *et al.* 1996), we considered the possibility that the suppression observed in the absence of Rpd3L is due to increased Sir spreading and dilution of the silencing at the HM loci. However, the suppression by *rph1*∆ and *rxt2-Q102STOP* was not prevented by *HML* deletion, nor was it haploid-specific (Figure S3), indicating that this is not a consequence of mating-type heterozygosity.

## Discussion

Using an unbiased approach, we found several pathways that contribute to the DNA damage sensitivity of *rad53*∆ mutants. Deletion of NMD and mating-type silencing made both *rad53*∆ and *RAD53* cells more resistant to DNA damage, and were therefore not characterized in detail (Figure 5 and Figure 6; Haber 2012). Loss of NMD has been shown to upregulate the levels of several proteins involved in homologous recombination and increase recombination rates (Janke *et al.* 2016), which could explain the suppression we have seen. Although the exact molecular mechanism by which *MAT* heterozygosity confers more resistance to DNA damage is unknown, some aspects of DNA repair are known to be under mating-type control; for example, nonhomologous end joining is repressed and spontaneous recombination is enhanced in *MAT***a**/α cells [reviewed in Haber (2012)].

Our results indicate that the DNA damage sensitivity of *rad53*∆ cells is partially suppressed by loss of the Rpd3L complex (Figure 1 and Figure 2). The loss of Rpd3L does not lead to hyperresistance to MMS in wild-type cells (Figure 5), indicating that Rpd3L specifically contributes to MMS sensitivity primarily in checkpoint-deficient cells. Moreover, suppression is not prevented by *HML* deletion, nor is it haploid-specific (Figure S3), thus ruling out *MAT* heterozygosity as an explanation for the suppression. Rpd3L has been shown to inhibit/delay the activation of > 100 replication origins in HU in *RAD53* cells (Knott *et al.* 2009). We cannot rule out the possibility that some deregulation of origin firing in the absence of Rpd3L may help suppress MMS sensitivity; however, origin firing is already deregulated in the absence of *RAD53*, and this deregulation does not make a major contribution to MMS survival (Zegerman and Diffley 2010). Indeed, deleting *RPD3* results in earlier replication of late origins (Vogelauer *et al.* 2002), similar to the effect of deleting *RAD53*. Rpd3 also plays an important role in gene regulation (Rundlett *et al.* 1996). However, new protein synthesis is not required for viability in HU (Tercero *et al.* 2003) or in MMS (Figure S4), arguing that suppression is not due to changes in gene expression.

The possibility we favor is that hyperacetylated chromatin somehow directly promotes replication fork stabilization. Previous data support a positive role for histone acetylation in fork progression (Choy and Kron 2002; Wurtele *et al.* 2012; Kurat *et al.* 2017), and the S-phase checkpoint has been suggested to facilitate DNA replication fork progression during replication stress by increasing chromatin accessibility around replication forks (Rodriguez and Tsukiyama 2013). Hyperacetylated chromatin might favor replication fork stabilization directly by easing fork progression though a more open chromatin or indirectly by the recruitment of some essential factor for fork stabilization. Alternatively, more accessible DNA might prevent unscheduled recombination at stalled forks or enable recombination-dependent fork restart. Although the consequences of histone acetylation to recombinational repair are currently unclear, it is well established that histone acetylation occurs at double-strand breaks (Bao 2011) and that a complex regulatory network of chromatin marks is involved in tuning recombination mechanisms (Clouaire and Legube 2015).

Rad53 might thus inhibit Rpd3L or Rph1, perhaps directly at stalled forks. We note that Rph1 has been shown to be a Rad53 target (Kim *et al.* 2002; Liang *et al.* 2011) and that its binding to chromatin has been shown to be inhibited by DNA damage (Liang *et al.* 2011), opening up the possibility that Rph1 is the essential Rad53 target for DNA damage survival. Among the many putative Rad53 phosphorylation sites in Rph1, Rph1-S652 has been suggested to be involved in the dissociation from chromatin upon UV irradiation (Liang *et al.* 2011). However, we have seen that the *rph1-S652A* phosphodefective mutant is not sensitive to MMS (Figure S2B) and that the phosphomimic *rph1-S652D* does not cause any suppression of the MMS sensitivity of *rad53*∆ (Figure S2B). Therefore, Rad53 phosphorylation of this site is not sufficient to explain the role of Rad53 in fork stabilization. We cannot rule out the possibility that redundant Rad53 phosphorylation sites in Rph1 are involved in this function or that other Rad53 targets in the Rpd3L complex are involved. Addressing this will require a better understanding of Rad53 substrates.

Given the correlation between tumorigenesis and mutations in the S-phase checkpoints (Bartkova *et al.* 2005; Gorgoulis *et al.* 2005), our observation that HDAC depletion leads to a suppression of lethality of yeast checkpoint mutants is in sharp contrast with the desired toxic effect for HDAC inhibitors in cancer. A large group of malignancies is associated with aberrant HDAC expression and activity, and many HDAC inhibitors have been shown to act synergistically with both chemo- and radiotherapy [reviewed in Kristensen *et al.* (2009)]. Paradoxically, and in line with our observations, HDAC inhibitors have also been shown to have a radiation-protective effect, as shown for the skin (Chung *et al.* 2004). Therefore, the mechanism of fork protection by histone acetylation both in normal and checkpoint-defective cells emerges as an interesting mechanism to be further explored.

## References

[bib1] Allen J. B. Zhou Z. Siede W. Friedberg E. C. Elledge S. J., 1994 The SAD1/RAD53 protein kinase controls multiple checkpoints and DNA damage-induced transcription in yeast. Genes Dev. 8: 2401–2415. 10.1101/gad.8.20.2401 7958905

[bib2] Bao Y., 2011 Chromatin response to DNA double-strand break damage. Epigenomics 3: 307–321. 10.2217/epi.11.14 22122340

[bib3] Bartkova J. Horejsi Z. Koed K. Kramer A. Tort F., 2005 DNA damage response as a candidate anti-cancer barrier in early human tumorigenesis. Nature 434: 864–870. 10.1038/nature03482 15829956

[bib4] Carrozza M. J. Florens L. Swanson S. K. Shia W. J. Anderson S., 2005 Stable incorporation of sequence specific repressors Ash1 and Ume6 into the Rpd3L complex. Biochim. Biophys. Acta 1731: 77–87; discussion 75–76 10.1016/j.bbaexp.2005.09.005 16314178

[bib5] Choy J. S. Kron S. J., 2002 NuA4 subunit Yng2 function in intra-S-phase DNA damage response. Mol. Cell. Biol. 22: 8215–8225. 10.1128/MCB.22.23.8215-8225.2002 12417725 PMC134065

[bib6] Chung Y. L. Wang A. J. Yao L. F., 2004 Antitumor histone deacetylase inhibitors suppress cutaneous radiation syndrome: implications for increasing therapeutic gain in cancer radiotherapy. Mol. Cancer Ther. 3: 317–325. 15026552

[bib7] Ciccia A. Elledge S. J., 2010 The DNA damage response: making it safe to play with knives. Mol. Cell 40: 179–204. 10.1016/j.molcel.2010.09.019 20965415 PMC2988877

[bib8] Cingolani P. Patel V. M. Coon M. Nguyen T. Land S. J., 2012 Using Drosophila melanogaster as a model for genotoxic chemical mutational studies with a new program, SnpSift. Front. Genet. 3: 35 10.3389/fgene.2012.00035 22435069 PMC3304048

[bib9] Clouaire T. Legube G., 2015 DNA double strand break repair pathway choice: a chromatin based decision? Nucleus 6: 107–113. 10.1080/19491034.2015.1010946 25675367 PMC4615830

[bib10] Cobb J. A. Schleker T. Rojas V. Bjergbaek L. Tercero J. A., 2005 Replisome instability, fork collapse, and gross chromosomal rearrangements arise synergistically from Mec1 kinase and RecQ helicase mutations. Genes Dev. 19: 3055–3069. 10.1101/gad.361805 16357221 PMC1315408

[bib11] De Piccoli G. Katou Y. Itoh T. Nakato R. Shirahige K., 2012 Replisome stability at defective DNA replication forks is independent of S phase checkpoint kinases. Mol. Cell 45: 696–704. 10.1016/j.molcel.2012.01.007 22325992

[bib12] Desany B. A. Alcasabas A. A. Bachant J. B. Elledge S. J., 1998 Recovery from DNA replicational stress is the essential function of the S-phase checkpoint pathway. Genes Dev. 12: 2956–2970. 10.1101/gad.12.18.2956 9744871 PMC317167

[bib13] Diffley J. F. X. Cocker J. H. Dowell S. J. Rowley A., 1994 Two steps in the assembly of complexes at yeast replication origins in vivo. Cell 78: 303–316. 10.1016/0092-8674(94)90299-2 8044842

[bib14] Dungrawala H. Rose K. L. Bhat K. P. Mohni K. N. Glick G. G., 2015 The replication checkpoint prevents two types of fork collapse without regulating replisome stability. Mol. Cell 59: 998–1010. 10.1016/j.molcel.2015.07.030 26365379 PMC4575883

[bib15] Foiani M. Marini F. Gamba D. Lucchini G. Plevani P., 1994 The B subunit of the DNA polymerase alpha-primase complex in *Saccharomyces cerevisiae* executes an essential function at the initial stage of DNA replication. Mol. Cell. Biol. 14: 923–933. 10.1128/MCB.14.2.923 8289832 PMC358447

[bib16] Friis J. Roman H., 1968 The effect of the mating-type alleles on intragenic recombination in yeast. Genetics 59: 33–36. 5683638 10.1093/genetics/59.1.33 PMC1211930

[bib18] Gerstung M. Beisel C. Rechsteiner M. Wild P. Schraml P., 2012 Reliable detection of subclonal single-nucleotide variants in tumour cell populations. Nat. Commun. 3: 811 10.1038/ncomms1814 22549840

[bib19] Gorgoulis V. G. Vassiliou L. V. Karakaidos P. Zacharatos P. Kotsinas A., 2005 Activation of the DNA damage checkpoint and genomic instability in human precancerous lesions. Nature 434: 907–913. 10.1038/nature03485 15829965

[bib20] Haber J. E., 2012 Mating-type genes and MAT switching in Saccharomyces cerevisiae. Genetics 191: 33–64. 10.1534/genetics.111.134577 22555442 PMC3338269

[bib21] Heude M. Fabre F., 1993 a/alpha-control of DNA repair in the yeast Saccharomyces cerevisiae: genetic and physiological aspects. Genetics 133: 489–498. 8454201 10.1093/genetics/133.3.489 PMC1205337

[bib22] Hu F. Alcasabas A. A. Elledge S. J., 2001 Asf1 links Rad53 to control of chromatin assembly. Genes Dev. 15: 1061–1066. 10.1101/gad.873201 11331602 PMC312686

[bib23] Huang M. Zhou Z. Elledge S. J., 1998 The DNA replication and damage checkpoint pathways induce transcription by inhibition of the Crt1 repressor. Cell 94: 595–605. 10.1016/S0092-8674(00)81601-3 9741624

[bib24] Janke C. Magiera M. M. Rathfelder N. Taxis C. Reber S., 2004 A versatile toolbox for PCR-based tagging of yeast genes: new fluorescent proteins, more markers and promoter substitution cassettes. Yeast 21: 947–962. 10.1002/yea.1142 15334558

[bib25] Janke R. Kong J. Braberg H. Cantin G. Yates J. R. III, 2016 Nonsense-mediated decay regulates key components of homologous recombination. Nucleic Acids Res. 44: 5218–5230. 10.1093/nar/gkw182 27001511 PMC4914092

[bib26] Joshi A. A. Struhl K., 2005 Eaf3 chromodomain interaction with methylated H3–K36 links histone deacetylation to Pol II elongation. Mol. Cell 20: 971–978. 10.1016/j.molcel.2005.11.021 16364921

[bib27] Kadosh D. Struhl K., 1998 Histone deacetylase activity of Rpd3 is important for transcriptional repression in vivo. Genes Dev. 12: 797–805. 10.1101/gad.12.6.797 9512514 PMC316629

[bib28] Kim E. M. Burke D. J., 2008 DNA damage activates the SAC in an ATM/ATR-dependent manner, independently of the kinetochore. PLoS Genet. 4: e1000015 10.1371/journal.pgen.1000015 18454191 PMC2265443

[bib29] Kim E. M. Jang Y. K. Park S. D., 2002 Phosphorylation of Rph1, a damage-responsive repressor of PHR1 in Saccharomyces cerevisiae, is dependent upon Rad53 kinase. Nucleic Acids Res. 30: 643–648. 10.1093/nar/30.3.643 11809875 PMC100300

[bib30] Kipling D. Tambini C. Kearsey S. E., 1991 Rar mutations which increase artificial chromosome stability in Saccharomyces cerevisiae identify transcription and recombination proteins. Nucleic Acids Res. 19: 1385–1391. 10.1093/nar/19.7.1385 2027746 PMC333890

[bib31] Klose R. J. Gardner K. E. Liang G. Erdjument-Bromage H. Tempst P., 2007 Demethylation of histone H3K36 and H3K9 by Rph1: a vestige of an H3K9 methylation system in Saccharomyces cerevisiae? Mol. Cell. Biol. 27: 3951–3961. 10.1128/MCB.02180-06 17371840 PMC1900024

[bib32] Knott S. R. Viggiani C. J. Tavare S. Aparicio O. M., 2009 Genome-wide replication profiles indicate an expansive role for Rpd3L in regulating replication initiation timing or efficiency, and reveal genomic loci of Rpd3 function in Saccharomyces cerevisiae. Genes Dev. 23: 1077–1090. 10.1101/gad.1784309 19417103 PMC2682954

[bib33] Kristensen L. S. Nielsen H. M. Hansen L. L., 2009 Epigenetics and cancer treatment. Eur. J. Pharmacol. 625: 131–142. 10.1016/j.ejphar.2009.10.011 19836388

[bib34] Kurat C. F. Yeeles J. T. Patel H. Early A. Diffley J. F., 2017 Chromatin controls DNA replication origin selection, lagging-strand synthesis, and replication fork rates. Mol. Cell 65: 117–130. 10.1016/j.molcel.2016.11.016 27989438 PMC5222724

[bib35] Labib K. De Piccoli G., 2011 Surviving chromosome replication: the many roles of the S-phase checkpoint pathway. Philos. Trans. R. Soc. Lond. B Biol. Sci. 366: 3554–3561. 10.1098/rstb.2011.0071 22084382 PMC3203454

[bib36] Liang C. Y. Hsu P. H. Chou D. F. Pan C. Y. Wang L. C., 2011 The histone H3K36 demethylase Rph1/KDM4 regulates the expression of the photoreactivation gene PHR1. Nucleic Acids Res. 39: 4151–4165. 10.1093/nar/gkr040 21296759 PMC3105397

[bib37] Longtine M. S. McKenzie A. III Demarini D. J. Shah N. G. Wach A., 1998 Additional modules for versatile and economical PCR-based gene deletion and modification in Saccharomyces cerevisiae. Yeast 14: 953–961. 10.1002/(SICI)1097-0061(199807)14:10<953::AID-YEA293>3.0.CO;2-U 9717241

[bib38] Lopes M. Cotta-Ramusino C. Pellicioli A. Liberi G. Plevani P., 2001 The DNA replication checkpoint response stabilizes stalled replication forks. Nature 412: 557–561. 10.1038/35087613 11484058

[bib39] Lopez-Mosqueda J. Maas N. L. Jonsson Z. O. Defazio-Eli L. G. Wohlschlegel J., 2010 Damage-induced phosphorylation of Sld3 is important to block late origin firing. Nature 467: 479–483. 10.1038/nature09377 20865002 PMC3393088

[bib40] Lucca C. Vanoli F. Cotta-Ramusino C. Pellicioli A. Liberi G., 2004 Checkpoint-mediated control of replisome-fork association and signalling in response to replication pausing. Oncogene 23: 1206–1213. 10.1038/sj.onc.1207199 14647447

[bib41] Maillet L. Boscheron C. Gotta M. Marcand S. Gilson E., 1996 Evidence for silencing compartments within the yeast nucleus: a role for telomere proximity and Sir protein concentration in silencer-mediated repression. Genes Dev. 10: 1796–1811. 10.1101/gad.10.14.1796 8698239

[bib43] Moriel-Carretero M. Aguilera A., 2010 A postincision-deficient TFIIH causes replication fork breakage and uncovers alternative Rad51- or Pol32-mediated restart mechanisms. Mol. Cell 37: 690–701. 10.1016/j.molcel.2010.02.008 20227372

[bib44] Quan T. K. Hartzog G. A., 2010 Histone H3K4 and K36 methylation, Chd1 and Rpd3S oppose the functions of Saccharomyces cerevisiae Spt4-Spt5 in transcription. Genetics 184: 321–334. 10.1534/genetics.109.111526 19948887 PMC2828714

[bib45] Rodriguez J. Tsukiyama T., 2013 ATR-like kinase Mec1 facilitates both chromatin accessibility at DNA replication forks and replication fork progression during replication stress. Genes Dev. 27: 74–86. 10.1101/gad.202978.112 23307868 PMC3553285

[bib46] Rundlett S. E. Carmen A. A. Kobayashi R. Bavykin S. Turner B. M., 1996 HDA1 and RPD3 are members of distinct yeast histone deacetylase complexes that regulate silencing and transcription. Proc. Natl. Acad. Sci. USA 93: 14503–14508. 10.1073/pnas.93.25.14503 8962081 PMC26162

[bib47] Rusche L. N. Kirchmaier A. L. Rine J., 2003 The establishment, inheritance, and function of silenced chromatin in Saccharomyces cerevisiae. Annu. Rev. Biochem. 72: 481–516. 10.1146/annurev.biochem.72.121801.161547 12676793

[bib48] Santocanale C. Diffley J. F. X., 1998 A Mec1- and Rad53-dependent checkpoint controls late-firing origins of DNA replication. Nature 395: 615–618. 10.1038/27001 9783589

[bib49] Santocanale C. Sharma K. Diffley J. F. X., 1999 Activation of dormant origins of DNA replication in budding yeast. Genes Dev. 13: 2360–2364. 10.1101/gad.13.18.2360 10500092 PMC317032

[bib50] Scott K. L. Plon S. E., 2003 Loss of Sin3/Rpd3 histone deacetylase restores the DNA damage response in checkpoint-deficient strains of Saccharomyces cerevisiae. Mol. Cell. Biol. 23: 4522–4531. 10.1128/MCB.23.13.4522-4531.2003 12808094 PMC164854

[bib51] Segurado M. Diffley J. F. X., 2008 Separate roles for the DNA damage checkpoint protein kinases in stabilizing DNA replication forks. Genes Dev. 22: 1816–1827. 10.1101/gad.477208 18593882 PMC2492668

[bib52] Shevchenko A. Roguev A. Schaft D. Buchanan L. Habermann B., 2008 Chromatin Central: towards the comparative proteome by accurate mapping of the yeast proteomic environment. Genome Biol. 9: R167 10.1186/gb-2008-9-11-r167 19040720 PMC2614481

[bib53] Shirahige K. Hori Y. Shiraishi K. Yamashita M. Takahashi K., 1998 Regulation of DNA-replication origins during cell-cycle progression. Nature 395: 618–621. 10.1038/27007 9783590

[bib57] Tercero J. A. Diffley J. F. X., 2001 Regulation of DNA replication fork progression through damaged DNA by the Mec1/Rad53 checkpoint. Nature 412: 553–557. 10.1038/35087607 11484057

[bib58] Tercero J. A. Longhese M. P. Diffley J. F. X., 2003 A central role for DNA replication forks in checkpoint activation and response. Mol. Cell 11: 1323–1336. 10.1016/S1097-2765(03)00169-2 12769855

[bib59] Valencia-Burton M. Oki M. Johnson J. Kamakaka R. T. Haber J. E., 2006 Different mating type-regulated genes affect the DNA repair defects in Saccharomyces RAD51, RAD52, and RAD55 mutants. Genetics 174: 41–55. 10.1534/genetics.106.058685 16782999 PMC1569815

[bib60] Van Driessche B. Tafforeau L. Hentges P. Carr A. M. Vandenhaute J., 2005 Additional vectors for PCR-based gene tagging in Saccharomyces cerevisiae and Schizosaccharomyces pombe using nourseothricin resistance. Yeast 22: 1061–1068. 10.1002/yea.1293 16200506

[bib61] Vogelauer M. Rubbi L. Lucas I. Brewer B. J. Grunstein M., 2002 Histone acetylation regulates the time of replication origin firing. Mol. Cell 10: 1223–1233. 10.1016/S1097-2765(02)00702-5 12453428

[bib62] Wurtele H. Kaiser G. S. Bacal J. St-Hilaire E. Lee E. H., 2012 Histone H3 lysine 56 acetylation and the response to DNA replication fork damage. Mol. Cell. Biol. 32: 154–172. 10.1128/MCB.05415-11 22025679 PMC3255698

[bib63] Zegerman P. Diffley J. F. X., 2009 DNA replication as a target of the DNA damage checkpoint. DNA Repair (Amst.) 8: 1077–1088. 10.1016/j.dnarep.2009.04.023 19505853

[bib64] Zegerman P. Diffley J. F. X., 2010 Checkpoint-dependent inhibition of DNA replication initiation by Sld3 and Dbf4 phosphorylation. Nature 467: 474–478. 10.1038/nature09373 20835227 PMC2948544

[bib65] Zeman M. K. Cimprich K. A., 2014 Causes and consequences of replication stress. Nat. Cell Biol. 16: 2–9. 10.1038/ncb2897 24366029 PMC4354890

[bib66] Zhao X. Muller E. G. Rothstein R., 1998 A suppressor of two essential checkpoint genes identifies a novel protein that negatively affects dNTP pools. Mol. Cell 2: 329–340. 10.1016/S1097-2765(00)80277-4 9774971

[bib67] Zhou J. Zhou B. O. Lenzmeier B. A. Zhou J. Q., 2009 Histone deacetylase Rpd3 antagonizes Sir2-dependent silent chromatin propagation. Nucleic Acids Res. 37: 3699–3713. 10.1093/nar/gkp233 19372273 PMC2699518

[bib68] Zhou Z. Elledge S. J., 1993 DUN1 encodes a protein kinase that controls the DNA damage response in yeast. Cell 75: 1119–1127. 10.1016/0092-8674(93)90321-G 8261511

